# Association of Active Human Herpesvirus-6, -7 and Parvovirus B19 Infection with Clinical Outcomes in Patients with Myalgic Encephalomyelitis/Chronic Fatigue Syndrome

**DOI:** 10.1155/2012/205085

**Published:** 2012-08-13

**Authors:** Svetlana Chapenko, Angelika Krumina, Inara Logina, Santa Rasa, Maksims Chistjakovs, Alina Sultanova, Ludmila Viksna, Modra Murovska

**Affiliations:** ^1^August Kirchenstein Institute of Microbiology and Virology, Riga Stradins University, Ratsupites Street 5, LV-1067 Riga, Latvia; ^2^Department of Infectology and Dermatology, Riga Stradins University, Linezera Street 3, LV-1006 Riga, Latvia; ^3^Department of Neurology and Neurosurgery, Riga Stradins University, Pilsonu Street 13, LV-1002 Riga, Latvia

## Abstract

Frequency of active human herpesvirus-6, -7 (HHV-6, HHV-7) and parvovirus B19 (B19) infection/coinfection and its association with clinical course of ME/CFS was evaluated. 108 ME/CFS patients and 90 practically healthy persons were enrolled in the study. Viral genomic sequences were detected by PCR, virus-specific antibodies and cytokine levels—by ELISA, HHV-6 variants—by restriction analysis. Active viral infection including concurrent infection was found in 64.8% (70/108) of patients and in 13.3% (12/90) of practically healthy persons. Increase in peripheral blood leukocyte DNA HHV-6 load as well as in proinflammatory cytokines' levels was detected in patients during active viral infection. Definite relationship was observed between active betaherpesvirus infection and subfebrility, lymphadenopathy and malaise after exertion, and between active B19 infection and multijoint pain. Neuropsychological disturbances were detected in all patients. The manifestation of symptoms was of more frequent occurrence in patients with concurrent infection. The high rate of active HHV-6, HHV-7 and B19 infection/coinfection with the simultaneous increase in plasma proinflammatory cytokines' level as well as the association between active viral infection and distinctive types of clinical symptoms shows necessity of simultaneous study of these viral infections for identification of possible subsets of ME/CFS.

## 1. Introduction

Myalgic encephalomyelitis/chronic fatigue syndrome (ME/CFS) is a disease characterized by profound disabling fatigue lasting at least 6 months and accompanied by a combination of nonspecific symptoms. According to the 1994 US Center's for Disease Control and Prevention (CDC) case definition, which at present, is widespread in research and clinical practice, at least four out of eight symptoms (impaired memory or concentration, sore throat, tender cervical or axillary lymph nodes, muscle pain, multi-joint pain, new headaches, sleep disturbances and post-exertion malaise) should be present in cases of ME/CFS [1]. During the clinical course of disease multiple body systems are affected by immune, neuroendocrine, musculoskeletal as well as psychiatric factors that reflect on the heterogeneity of the disease. Because fatigue is a common symptom of many diseases, a wide differential diagnosis needs to be done. The observation that many cases of the disease begin with a flu-like illness has prompted the hypothesis that viral infections are implicated in this disorder. 

Infections of human *β*-herpesviruses-human herpesvirus-6 and -7 (HHV-6, HHV-7), cytomegalovirus (CMV) [2–4], Epstein-Barr virus [5, 6], parvovirus B19 (B19) [7–9], and enterovirus [10] are suggested as etiological agents for ME/CFS. The major hypothesis of the pathogenesis of the disease is that persistent viral infections may trigger and lead to chronic activation of the immune system with abnormal regulation of cytokine production [11]. However, until now the role of viral infections as etiological agents for ME/CFS has been evaluated inconsistently [12–15]. Identification of specific biomarkers for differential diagnosis of ME/CFS has intensively been studied [16–19]. 

HHV-6 and HHV-7 are lymphotropic, neurotropic, and immunomodulating viruses which primary infection is followed by lifelong persistency. Reactivation of viruses can provoke the development of abnormalities involving the immune system and nervous system and probably may trigger ME/CFS [14]. Two distinct variants of HHV-6 (A and B) have been described [19] and the human glycoprotein CD46 has been recorded as a receptor molecule for both variants [20]. Clinical features of HHV-6A infection remain unclear. 

Human parvovirus B19 was first discovered by Cossart et al. [21] in the sera of healthy blood donors. The virus is ubiquitous and course of infection depends on the host's hematological status and immune response. Cellular receptor for B19 is globoside (blood group P antigen) [22]. Tissue distribution of P antigen may explain the clinical manifestation of the viral infection [23]. 

The appearance of antibodies to B19 is associated with clearance of the virus from the bloodstream; however, the high frequency of persistence of B19 DNA in tissue of healthy persons under the presence of anti-B19 antibodies indicates that complete eradication of the virus from host body has not occurred [24]. It is unclear how often ME/CFS appears after B19 infection and whether B19 viremia or other factors cause ME/CFS [9].

*The aim* of this study was to determine frequency of active HHV-6, HHV-7, and B19 infection/coinfection and to evaluate association of active single and concurrent infection with clinical outcomes in cases of ME/CFS.

## 2. Materials and Methods 

### 2.1. Patients

One hundred eight randomly selected patients (66 females, 42 males; mean age 37 years) with clinically diagnosed ME/CFS after rigorous examination of criteria (fatigue for at least six months and at least four of eight symptoms: postexertional malaise, impaired memory or concentration, unrefreshing sleep, muscle pain, multijoint pain, tender cervical or axillary lymph nodes, sore throat, headache) corresponding to the 1994 CDC definition and 90 practically healthy persons (55 females, 35 males; mean age 39 years) were investigated for evidence of HHV-6, HHV-7 and B19 infection/coinfection. The presence and frequency of clinical features in ME/CFS patients were examined in relation to active viral infection. Severity of fatigue was evaluated by Fatigue Severity Scale (FSS) with maximal score of 72 points [25]. The cohort was established with the approval of the Ethics Committee of the Riga Stradins University and all participants gave their informed consent prior to the examination. Ninety practically healthy persons (55 females, 35 males; mean age 39 years) were included in this examination as a control group. 

### 2.2. HHV-6 and B19 Serology

Plasma samples were tested by ELISA kits (Panbio, Sinnamon Park, OLD, Australia) for specific anti-HHV-6 IgM and IgG class antibodies according manufacturer's protocols. Tests for antibodies against B19 were carried out using B19 IgG and IgM the anti-VP2 enzymatic immunoassay kits (Biotrin, Dublin, Ireland) in accordance with the manufacturer's recommendations.

### 2.3. Nested Polymerase Chain Reaction (nPCR)

The technique of nPCR was used to detect viral genomic sequences in DNA isolated from peripheral blood leukocytes (PBLs) and cell free plasma (markers of persistent and active infection, resp.). Total DNA was isolated from 0.5 ml of fresh whole blood by phenol-chloroform extraction. The QIAamp Blood Kit (QIAGEN, Hilden, Germany) was used to purify DNA from 200 *μ*L of cell-free blood plasma. The plasma samples were treated with DNase I (Fermentas, Vilnius, Lithuania) before DNA purification. To assure the quality of the PBL DNA and to exclude contamination of plasma DNA by cellular DNA, PCR was performed with primers that recognized the globin gene. PCR amplification of viral DNA was carried out in the presence of 1 *μ*g of PBL DNA or 10 *μ*L of plasma DNA (which corresponded to 100 *μ*L of plasma). HHV-6, HHV-7, and B19 DNA were detected in accordance with Secchiero et al. [26], Berneman et al. [27], and Cavallo et al. [28], respectively. Positive controls (HHV-6 and HHV-7 genomic DNA; Advanced Biotechnologies Inc, Columbia, MD, USA and B19 genomic DNA isolated from viremic serum kindly provided by Prof. K.Hedman, Department of Virology, Heartman Institute, University of Helsinki) and negative controls (DNA obtained from practically healthy HHV-6, HHV-7 and B19 negative blood donor and no template DNA) were included in each experiment. 

Criteria to define persistent viral infection were the presence of virus-specific IgG class antibodies in blood plasma and viral genomic sequences in DNA isolated from the whole blood. The presence of HHV-6-specific IgM class antibodies in blood plasma and viral genomic sequence in plasma DNA samples, as well as elevated titer of virus-specific IgG class antibodies, without IgM antibodies and viral genomic sequence in plasma DNA samples was defined as active HHV-6 infection. The cases with HHV-7-specific sequence in DNA isolated from cell free blood plasma were defined as active HHV-7 infection cases. The presence of HHV-7-specific antibodies was not examined due to the lack of commercial test systems for IgM class antibody detection. The cases with persistent infection and without markers of active viral infection were defined as latent stage of persistent infection (latent infection) cases. B19 genomic sequence in plasma DNA samples with or without the presence of IgM class specific antibodies and the presence of viral sequence in PBL DNA samples together with IgM class specific antibodies in blood plasma we defined as active B19 infection.

### 2.4. Quantitative Real-Time PCR

The viral load of HHV-6 in PBL DNA samples from patients with latent and active viral infections was determined using the HHV-6 Real-Time Alert Q-PCR kit (Nanogen Advanced Diagnostics, Buttigliera Alta, Italy) and an Applied Biosystems 7500 Real-time PCR System (Applied Biosystems, Carlsbad, CA, USA), in accordance with the manufacturer's recommendations.

### 2.5. Restriction Endonuclease Analysis

Restriction endonuclease analysis was carried out using the restriction enzyme* Hind*III (Fermentas, Vilnius, Lithuania), which cleaves the 163 bp HHV-6B amplimer into two fragments of 66 and 97 bp but does not cleave HHV-6A amplimer. 

### 2.6. Assay for Cytokine Determination

Endogen Human ELISA kits (Pierce Biotechnology, Rockford, IL, USA) were used to detect the level of tumor necrosis factor (TNF)-*α*, interleukin (IL)-6 and IL-4 in plasma samples from ME/CFS patients according to the manufacturer's recommendations. The sensitivity of the ELISAs was 2 pg/mL for TNF-*α* and <1 pg/mL for IL-6, and <4 pg/mL for IL-4. All samples were tested in duplicate. Plasma samples were processed immediately after collection and then stored at −70°C.

### 2.7. Statistical Analysis

Statistical difference in the frequency of active HHV-6, HHV-7, and B19 infection/coinfection between tested groups was assessed by Odds ratio, 95% CI values and Fisher's exact test and in the levels of cytokines-by Student's *t*-test, using MedCalc software for Windows, version 12.2.1; a value of *P* < 0.05 was considered to be significant.

## 3. Results

### 3.1. HHV-6 and B-19 Serology

Specific anti-HHV-6 antibodies were detected in 87/108 (80.6%) plasma samples (IgG-71, IgM-3, IgM + IgG-13) from the ME/CFS patients versus 69/90 (76.7%) practically healthy persons' plasma samples (IgG-67, IgM + IgG-2) and specific anti-B19 antibodies in 92/108 (85.2%) plasma samples (IgG-62, IgM-6, IgM + IgG-24) from the ME/CFS patients versus 55/90 (61.1%) plasma samples ( IgG-44, IgM-2, IgM + IgG-9) from the practically healthy persons. 

### 3.2. Prevalence of Active HHV-6, HHV-7, and B19 Infections

Active viral infection/coinfection was detected in 70 (64.8%) and latent—in 32 (29.6%) out of 108 patients with ME/CFS. Six (5.6%) patients were negative for viral infection (Table 1). The rate of active viral infection was significantly higher in patients comparing to the rate in the practically healthy persons (70/108 and 12/90, resp.; Odds ratio 0.14, 95% CI 0.07–0.26, *P* = 0.0001). In the patients significant difference was detected between the frequency of single active and concurrent active viral infection (41/70, 58.6% and 29/70, 41.4%, resp.; Odds ratio 2.0, 95% CI 1.02–3.92, *P* = 0.044). Among the patients who were infected with a single virus, the rate of HHV-7 active infection (40%) was significantly higher in comparison with B19 (15.7%, Odds ratio 3.58, 95% CI 1.60–7.97, *P* = 0.002) and HHV-6 active infection (2.9%, Odds ratio 22.7, 95% CI 5.13–100.1, *P* < 0.0001). No significant difference was detected between the frequency of active concurrent dual HHV-6 + HHV-7 and dual HHV-7 + B19 infection (Odds ratio 0.61, 95% CI 0.25–1.47, *P* = 0.273). 

HHV-6B was identified in 15 and HHV-6A in one out of 16 PBL and plasma DNA samples.

### 3.3. HHV-6 DNA Load in PBL DNA Samples 

The number of HHV-6 DNA copies in PBL DNA of the ME/CFS patients with and without HHV-6 viremia was compared. A clear increase of HHV-6 load in PBL DNA was detected in 16 patients with plasma viremia in comparison with seven patients without it (132.61 ± 41.38 × 10^3^ and 8.73 ± 3.96 × 10^3^ copies/*μ*g DNA, resp.). 

### 3.4. Relationship between Plasma Level of TNF-*α*, IL-6, and IL-4 and Active Viral Infection/Coinfection

To investigate the relationship between the active viral infections and plasma cytokine levels, the levels of proinflammatory (TNF-*α*, IL-6) and anti-inflammatory (IL-4) cytokines were measured in 106 ME/CFS patients (Table 2). The mean levels of TNF-*α* and IL-6 cytokines were significantly higher in patients with active viral infection/coinfection (52.51 ± 15.13 pg/mL, 18.59 ± 3.56 pg/mL, resp.) than in those with latent (18.81 ± 2.52 pg/mL, 2.56 ± 1.02 pg/mL, resp.; *P* < 0.0001) or without viral infections (7.71 ± 3.07 pg/mL, 1.32 ± 3.07 pg/mL, resp.; *P* < 0.0001). No significant difference was detected between expression levels of TNF-*α* in the patients with active single HHV-7 and active single B19 infection, as well as with dual active HHV-6 + HHV-7 and dual active HHV-7 + B19 coinfection (Table 2).

The highest level of TNF-*α* was detected in patients with active triple HHV-6+HHV-7 + B19 coinfection. Significantly higher level of IL-6 expression was detected in plasma samples of the patients with single active B19 infection in comparison with the patients with single active HHV-7 infection (*P* < 0.001) (Table 2). The mean levels of this cytokine were also significantly higher in patients with active concurrent HHV-7 + B19, as well as in patients with triple HHV-6 + HHV-7 + B19 infection (29.19 ± 6.26 pg/mL, *P* < 0.001) in comparison with the level in patients with dual HHV-6 + HHV-7 infection (11.22 ± 3.14 pg/mL). None of the ME/CFS patients had increased plasma level of IL-4.

### 3.5. Assessment of Active Betaherpesviruses and B19 Infection/Coinfection in Association with Clinical Outcomes in ME/CFS Patients

Severe chronic fatigue for at least six months or longer was observed in all patients irrespective of the causation of the active infection (total FSS scores 58.89–60.99, *P* < 0.05). Subfebrility, tender cervical or axillary lymph nodes, and postexertional malaise were not revealed in the patients with single B19 active infection but were detected in patients with single HHV-7 active (50%, 75%, 100%, resp.), dual HHV-6 + HHV-7 (70%, 80%, 90%, resp.) as well as triple HHV-6 + HHV-7 + B19 coinfection (74.1%, 68.4%, 74.1%) (Table 3).

Although muscle pain was observed in all patients, the frequency of multijoint pain was more clearly displayed in all patients with active B19 infection, as in cases of single as well as in cases of coinfection with *β*-herpesviruses.

Severe postexertional malaise corresponding to “Exercise brings on my fatigue” by FSS was detected in all patients (mean score 6.94 ± 0.243 from 7 maximum) with single HHV-6, HHV-7, and in 9/10 with dual HHV-6 + HHV-7 coinfection as well as in 14/19 with triple HHV-6 + HHV-7 + B19 coinfection (90% and 74%, resp.).

Neuropsychological disturbances were observed in all 70 patients. Impaired memory was detected in 22 out of 57 (38.6%) patients with active *β*-herpesviruses infection/coinfection but not observed in patients with single HHV-6 and single B19 infection. Impaired concentration was detected in 34 out of 70 patients (48.6%), more frequently in patients with B19 infection. Sleep disturbances were revealed in 49 out of 70 (70%) patients, the sleepiness was more characteristic for patients with single HHV-6, B19, and dual HHV-7+B19 coinfection (2/2, 11/11 and 15/15, resp.).

Headaches of new type were observed in 16 out of 30 (53.3%) patients with B19 infection/coinfection and in 14 out of 38 (36.8%) patients with HHV-7 and dual HHV-6 + HHV-7 infection.

Chronic fatigue (for at least 6 months or longer period) was observed also in all 38 patients with latent infection and without infection (32 with latent infection and 6 without infection). Postexertional malaise (23/38, 60.5%, mean score 5.23 ± 0.135 from 7 maximum), impaired memory (34/38, 89.5%), decreased concentration (32/38, 84.2%), and sleep disturbances (24/38, 63.2%) were predominant symptoms in these patients. Subfebrility (10/38, 26.3%) and lymphadenopathy (11/38, 29%) were observed only in patients with latent single HHV-7 and dual HHV-6 + HHV-7 infection/coinfection; muscle pain (14/38, 36.8%) in patients with *β*-herpesviruses infection/coinfection (11/14) as well as with B19 infection/coinfection (3/14). Multijoint pain was observed in 15 out of 38 (39.5%) patients and in 10 of them B19 infection/coinfection was found. Headaches of new type were observed in 13 out of 38 (34.2%) patients. Among these 13 patients *β*-herpesviruses infection/coinfection was detected in 9 patients and B19 infection/coinfection in 4 patients. Clinical manifestations of the above-mentioned symptoms were not severe (total FSS scores were 42.83–48.90, *P* < 0.05). 

Subfebrility, lymphadenopathy, malaise after exertion, muscle pain, multijoint pain, sleep disturbances, and headaches of new type were more frequent in patients with active viral infection/coinfection than in patients with latent infection and without infection. Whereas presence of impaired memory and impaired concentration was more frequent in patients with latent infection and without infection in comparison to the patients with active infection. 

## 4. Discussion

ME/CFS is heterogeneous disorder with common set of symptoms that follows a viral infection. Despite efforts in the development of standardized research criteria to define ME/CFS [1, 11, 29, 30] progress in diagnosis and elucidation of the role of viral infections is slow, due to a lack of common standard clinical definition and specific biomarkers of disease. 

This study is the continuation of research of HHV-6 and HHV-7 as triggering factors for ME/CFS [3]. In the study reported herein, we investigated the frequency of active HHV-6, HHV-7 and parvovirus B19 infection/coinfection and potential relationship of active viral infection with different clinical symptoms in 108 patients with clinically diagnosed ME/CFS. 

The results of our study showed a high rate of HHV-6 and B19 seroprevalence among our patients and practically healthy persons. The positive rates of anti-HHV-6 and anti-B19 IgG class antibodies are similar in ME/CFS patients and practically healthy persons. At the same time, the elevated frequency of anti-HHV-6 IgM class antibodies is found in ME/CFS patients which is in concordance with previously observation by Patnaik et al. [31] and Ablashi et al. [2]. In contrast, Koelle et al. [32] and Cameron et al. [15] do not support these data. Also the elevated frequency of anti-B19 IgM class antibodies is found in ME/CFS patients in comparison with the practically healthy persons (Odds ratio 0.36, 95% CI 0.17–0.77, *P* = 0.009). High rates of active viral infection/coinfection in ME/CFS patients might be associated with insufficiency of the humoral immune response to the viruses. 

We identified active viral infection in 70 (65%) ME/CFS patients, 41 of them had active single HHV-6, HHV-7, and B19 infection and 29-concurrent (dual HHV-6 + HHV-7, HHV-7 + B19 and triple HHV-6 + HHV-7 + B19) infection. The active single HHV-7 and B19 infection, but not active single HHV-6 or active concurrent infection, has been previously detected in Latvian blood donors [33]. The active HHV-6 infection was confirmed by the detection of viral sequence in plasma DNA samples and a concomitant increase of HHV-6 load in PBLs. This corresponds with the data demonstrated by Ihira et al. [34]. 

Although HHV-6A is predominant in ME/CFS patients, it was detected in only one patient that might be limited to persistency sites other than the peripheral blood [8]. 

Despite the fact that the precise mechanism by which betaherpesviruses and B19 impair immunological function are not completely clear, previous investigations have shown that these viruses are effective modulators of the immune response, mainly by modulating the production of proinflammatory cytokines, including TNF-*α* and IL-6 [35, 36]. We detected the presence of significantly higher levels of TNF-*α* and IL-6 in plasma samples from patients with active viral infection. This finding confirms the immunomodulating properties of these viruses and is in concordance with the data of Fletcher et al. [37]. However, our data do not corroborate with the findings of Vollmer-Conna et al. [38] that have found no significant difference of cytokine production in patients with postinfection fatigue syndrome.

The relationship between active *β*-herpesviruses and B19 infection/coinfection and ME/CFS clinical symptoms is not clear. In the present study, clinical features of the disease were particularly studied in detail specially in subgroups of 70 patients with different active viral infections although clinical diagnosis of ME/CFS was confirmed in the presence of four from eight analyzed symptoms [1] in all patients including those with latent viral infection and without infection. The analysis of our results showed definite relationship between active single HHV-6 and HHV-7 infection and presence of subfebrility, lymphadenopathy, and malaise after exertion, and single B19 infection with multijoint pain. By concurrent infection no clear differences in manifestations of the symptoms were detected. Neuropsychological disturbances were detected in all ME/CFS patients. Neuropsychological disturbances were detected in all ME/CFS patients.

Results of our study correspond to the newest International Consensus Criteria for clinical case definition [30] which proposed several subsets of myalgic encephalomyelitis such as neurological, immune, metabolism/cardiorespiratory. 

## 5. Conclusions

The association between occurrence of ME/CFS clinical symptoms, HHV-6, HHV-7 and B19 infection/coinfection reactivation and increased expression levels of TNF-*α* and IL-6 allows suggesting that these immunomodulating pathogens are involved in ME/CFS etiopathogenesis. Their role as trigger factors could not be excluded. The correlation of distinctive active viral infection with various types of clinical symptoms shows necessity of simultaneous study of these viral infections for identification of possible subsets of ME/CFS.

## Figures and Tables

**Table 1:** Frequency of Active HHV-6, HHV-7 and B19 Infection in ME/CFS Patients and Practically Health Persons.

Viral infection	Plasma samples (*n* = 108)	Practically health persons (*n* = 90)
*Active viral infection *	70/108	12/90
Single HHV-6	2/108	0/90
Single HHV-7	28/108	10/90
Single B19	11/108	2/90
Dual HHV-6 + HHV-7	10/108	0/90
Dual HHV-7 + B19	15/108	0/90
Triple HHV-6 + HHV-7 + B19	4/108	0/90
*Latent viral infection *	32/108	53/90
*Without viral infection *	6/108	25/90

**Table 2:** Plasma cytokine levels in patients with ME/CFS.

Viral infection	TNF-alpha (pg/mL)	IL-6 (pg/mL)	IL-4 (pg/mL)
*Without (* *n* = 6)	7.71 ± 3.07	1.32 ± 0.43	<2
*Latent (n* = 32)	18.81 ± 2.52	2.56 ± 1.02	<2
*Active (n* = 68)
Single HHV-7 (*n* = 28)	44.81 ± 10.56	7.76 ± 2.45	<2
Single B19 (*n* = 11)	44.12 ± 11.67	15.62 ± 4.63	<2
Dual HHV-6 + HHV-7 (*n* = 10)	50.38 ± 8.74	11.22 ± 3.14	<2
Dual HHV-7 + B19 (*n* = 15)	49.89 ± 16.1	28.70 ± 3.05	<2
Triple HHV-6 + HHV-7 + B19 (*n* = 4)	73.33 ± 18.76	29.67 ± 4.52	<2

**Table 3:** Symptoms of ME/CFS in patients with active viral infection/coinfection.

Defining symptoms	Active viral infection				
	HHV-6 (*n* = 2)	HHV-7 (*n* = 28)	B19 (*n* = 11)	HHV-6 + HHV-7 (*n* = 10)	HHV-7 + B19 and HHV-6 + HHV-7 + B19 (*n* = 19)
Subfebrility	0/2	14/28	0/11	7/10	14/19
Lymphadenopathy	2/2	21/28	0/11	8/10	10/19
Malaise after exertion	2/2	28/28	0/11	9/10	14/19
Muscle pain	2/2	28/28	11/11	10/10	19/19
Multijoint pain	2/2	7/28	11/11	10/10	19/19
Impaired memory	0/2	7/28	0/11	6/10	9/19
Impaired concentration	2/2	7/28	11/11	4/10	10/19
Sleep disturbances	2/2	7/28	11/11	10/10	19/19
New headaches	0/2	14/28	11/11	0/10	5/19
